# The Coags Uncomplicated App: Fulfilling Educational Gaps Around Diagnosis and Laboratory Testing of Coagulation Disorders

**DOI:** 10.2196/mededu.6858

**Published:** 2017-04-18

**Authors:** Craig Kessler, Ellinor I Peerschke, Meera B Chitlur, Roshni Kulkarni, Natalia Holot, David L Cooper

**Affiliations:** ^1^ Georgetown University Medical Center Washington, DC United States; ^2^ Memorial Sloan Kettering Cancer Center New York, NY United States; ^3^ Carman and Ann Adams Department of Pediatrics Children's Hospital of Michigan Wayne State University Detroit, MI United States; ^4^ Michigan State University East Lansing, MI United States; ^5^ Novo Nordisk Inc. Plainsboro, NJ United States

**Keywords:** blood coagulation disorders, smartphone, diagnosis, hematology, differential diagnosis

## Abstract

**Background:**

Patients with coagulation disorders may present to a variety of physician specialties; however, accurate and efficient diagnosis can be challenging for physicians not specialized in hematology, due to identified gaps in knowledge around appropriate laboratory assays and interpretation of test results. Coags Uncomplicated was developed to fill this unmet educational need by increasing practical knowledge of coagulation disorders among nonexpert physicians and other health care professionals (HCPs) in a point-of-care (POC) setting.

**Objective:**

The aim of this study was to assess patterns of use of the mobile app Coags Uncomplicated, a tool designed to support education regarding accurate and efficient diagnosis of bleeding disorders.

**Methods:**

App metrics were obtained by tracking registered user data. Additionally, a survey was distributed to registered users, to assess circumstances and frequency of use.

**Results:**

The most common specialties of 7596 registered US users were hematology-oncology (n=1534, 20.19%), hematology (n=1014, 13.35%), and emergency medicine (n=1222, 16.09%); most identified as physicians (n=4082, 53.74%). Specialties accounting for the greatest numbers of screen views were hematology-oncology (99,390 views), hematology (47,808 views), emergency medicine (23,121 views), and internal medicine (22,586 views). The most common diagnostic endpoints reached were disseminated intravascular coagulation (DIC; 2713 times), liver disease effect (2108 times), and vitamin K deficiency (1584 times). Of 3424 users asked to take the survey, 262 responded (7.65%); most were physicians in direct clinical care (71%) and specialized in hematology-oncology (39%) or emergency medicine (21%). Most frequent use was reported by hematologists (69%, ≥6 times) and hematologists-oncologists (38%, ≥6 times). Most physicians (89.2%) reported using the app for patient-case-related education around appropriate use of laboratory tests in diagnostic evaluation. Physicians rated Lab Value Analyzer (mean 4.43) and Lab Test Algorithm (mean 4.46) tools highly on a 5-point “how helpful” scale and were likely to recommend the app to colleagues.

**Conclusions:**

App use among physicians and other HCPs is consistent with value as a POC educational tool, which may facilitate differential diagnoses and appropriate early consultation with hematologists.

## Introduction

### Background

For many physicians who first encounter patients with severe bleeding symptoms, the potential contribution of an underlying bleeding disorder may often be overlooked. When presented with an acutely bleeding patient, the focus of many primary care physicians, physicians working in the emergency and hospital settings, and physician trainees is “where” rather than “why” the patient is bleeding, and how to best manage the symptoms at hand. Underlying bleeding disorders are perceived to be rare; however, approximately 1% of individuals in the United States have von Willebrand disease (VWD) [1,2], approximately 1 in 5000 males is born with hemophilia [3], and many individuals have iatrogenic bleeding problems from medications. Furthermore, obtaining a rapid and accurate bleeding disorder diagnosis is critical for understanding patients’ long-term bleeding risks and management implications.

Bleeding disorders encompass a large number of unique conditions that require specialized knowledge and a stepwise strategy for accurate and efficient diagnosis, and they may be difficult for nonhematologists to diagnose. Many of these knowledge gaps were demonstrated through administration of a large survey to practicing physicians from various specialties, which presented a hypothetical case scenario of a patient with acquired hemophilia, a rare bleeding disorder [4]. Nonhematologists in this study were found to lack appropriate consideration of and response to bleeding symptoms and awareness of how to correctly interpret laboratory results as simple as an isolated prolonged activated partial thromboplastin time (aPTT) and to be hesitant in consulting with a hematologist once abnormal findings were identified. Furthermore, a particularly challenging task for nonhematologists is to understand the differential diagnostic considerations needed to distinguish among coagulation disorders with a similar set of bleeding patterns, such as qualitative platelet function disorders and VWD. Symptoms of platelet function disorders and VWD typically include nonspecific mucocutaneous bleeding symptoms such as epistaxis, menorrhagia (also called heavy menstrual bleeding), gingival bleeding, and easy bruising, which may present to physicians of a variety of specialties [5]. Physicians without expertise in hematology may stop evaluation after seeing a normal prothrombin time (PT), aPTT, and platelet count, rather than performing additional assessments needed to diagnose these disorders.

### Coags Uncomplicated App

For physicians faced with acutely bleeding patients, education regarding specific laboratory tests, interpretation of results, and potentially applicable diagnoses may be valuable in supporting early referral and initiation of treatment. A potentially important tool to increase awareness of important diagnostic considerations is mobile technology, as mobile devices are emerging as a useful platform for health care professionals (HCPs) to quickly access medical information, including traditional sources such as medical textbooks, professional society guidelines, drug references, and institution-specific therapy standards, as well as Web-based tools and mobile phone apps [6]. The Coags Uncomplicated app is a freely available educational tool that was developed as a collaboration between nationally recognized hematologists, coagulation laboratory experts, and an industry partner (Novo Nordisk Health Care AG), and it provides targeted, case-based education around the differential diagnosis of bleeding disorders. The app consists of 5 separate tools: Lab Value Analyzer (users input laboratory values and receive a list of potential conditions for differential diagnosis, and can click through to view educational materials about each disorder), Lab Test Algorithm (a step-by-step guide on laboratory assays with educational information regarding the interpretation of test results and important caveats about variables which affect test results), Neonatal Module (normative laboratory value lookups and laboratory testing algorithms based on gestational age), Face a Case (a review of interesting cases for users to apply their knowledge), and Coag Challenge (a timed quiz in which users can compete for rankings).

Here, we present information regarding physician and other-HCP use of Coags Uncomplicated and assess its value as an educational tool. Data include results from a survey of Coags Uncomplicated users, as well as app tracking metrics with data collected from actual app use, to assess real-world patterns of use over time.

## Methods

### App Development

The concept for the initial gap assessment leading to the development of Coags Uncomplicated came from hematologist advisors in 2009 and was crystallized in a case study that became the focus of a quantitative Internet survey and subsequent qualitative interviews conducted in early 2010 [4]. The first generation app platform was developed in the fall of 2010 in an iterative, collaborative process involving external experts (CMK, EIP) and Novo Nordisk Inc (DLC) with additional medical support from an agency that built the app (Cline Davis & Mann Inc, Princeton, NJ, USA).

The initial focus of the app was on case scenarios presenting with abnormal PT or a PTT test results, for which published diagnostic algorithms could be adapted toward a primary care or first responder (nonspecialist) audience by eliminating common disorders earlier in the algorithm (eg, liver failure, disseminated intravascular coagulation [DIC], and vitamin K deficiency bleeding). This process identified 30 diseases for which educational content was developed and 26 coagulation laboratory tests associated with the diagnosis of these disorders. Content around all disorders and tests was referenced to 79 sources and comprised 270 app scrolling screens occupying 623 distinct screenshots. Important diagnostic caveats were included, and emphasis was placed on the need for expert consultation in the ultimate differential diagnosis. The app was launched in December 2010 (United States) and July 2011 (global), and included 4 separate tools: the Lab Test Algorithm (graphically described in Multimedia Appendices 1-3), Lab Value Analyzer, Face a Case, and Coags Challenge.

Following initial launch of the app, feedback from hematologists identified additional specific needs around (1) neonatal bleeding disorder differential diagnoses and challenges associated with gestational age-adjusted “normal” laboratory values and (2) VWD, platelet function disorders, and other disorders associated with normal PT and aPTT results and often normal platelet counts. Because these disorders were associated with fewer published algorithms and wider variability in diagnostic approaches, additional experts were engaged (MBC, RK) in developing the second-generation platform. The expanded scope of this platform included educational materials on a total of 66 diseases and 34 laboratory tests supported by 193 references and comprised 583 scrollable screens captured by 1733 screenshots. This version included the Neonatal Module as well as additional Lab Test Algorithms (see Multimedia Appendices 4-6).

### App Tracking Metrics

In order to better understand the usage patterns of the app and to guide further content development, in the United States, registration was required before using the app. App tracking metrics describing patterns of use from the initial launch in December 2010 through February 2016 were obtained. Data include total numbers of screen views (by user specialty and by app tool) and the most frequent diagnostic endpoints reached. App users outside of the United States were not required to register.

### Survey Data

Initial tracking metrics demonstrated greater use of the app by hematology and hematology-oncology specialists than by nonspecialists. To better understand whether specialist app use was primarily for education or teaching or for case-based education and whether app use differed between hematology specialists and nonspecialists, a Web-based survey was developed. Participants were recruited from a database of 3424 registered (US-based) app users who had downloaded the app (version 1.0 or 2.0), and they were screened to ensure that they had used the app at least once. Participants were required to be adults (at least 18 years of age), and they had to have completed the survey between October 1 and October 11, 2013. Survey questions provided an assessment of respondent demographics and frequency of app use (including use for education to support actual patient cases) and ratings of app helpfulness and likelihood to recommend the app. Helpfulness was rated on a 5-point scale from 1 to 5 with 5=very helpful. Likelihood to recommend the app was rated on a 5-point scale from 1=not at all to 5=very likely.

## Results

### App Tracking Metrics

As of February 2016, the most common specialties listed by 7596 registered users included hematology-oncology (n=1534, 20.19%), hematology (n=1014, 13.35%), and emergency medicine (n=1222, 16.09%; Table 1). A majority of users identified themselves as doctors of medicine (MDs; n=4082, 53.74%); other common degrees or positions were doctors of osteopathy (DOs; n=364, 4.79%), registered nurses (RNs; n=639, 8.41%), nurse practitioners (NPs; n=415, 5.46%), doctors of pharmacy (PharmDs; n=283, 3.73%), and physician assistants (PAs; n=224, 2.95%).

**Figure 1:**
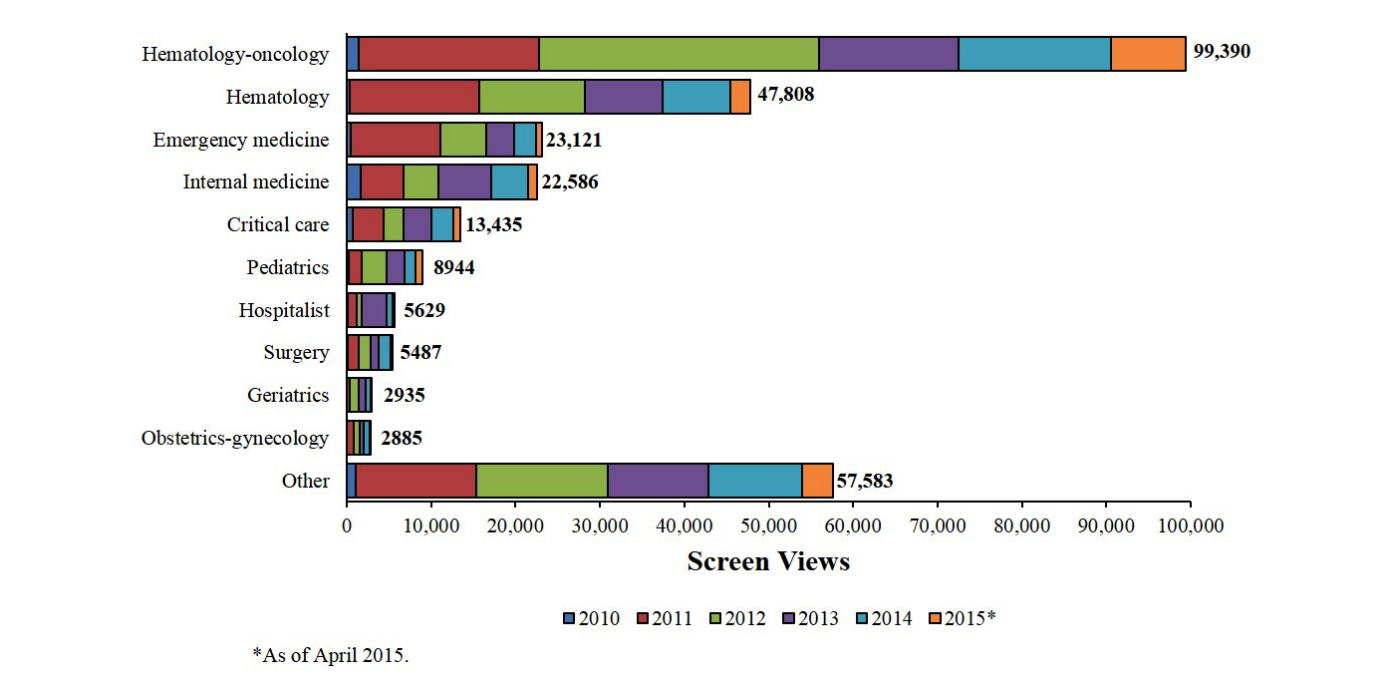
Screen views by specialty.

**Figure 2:**
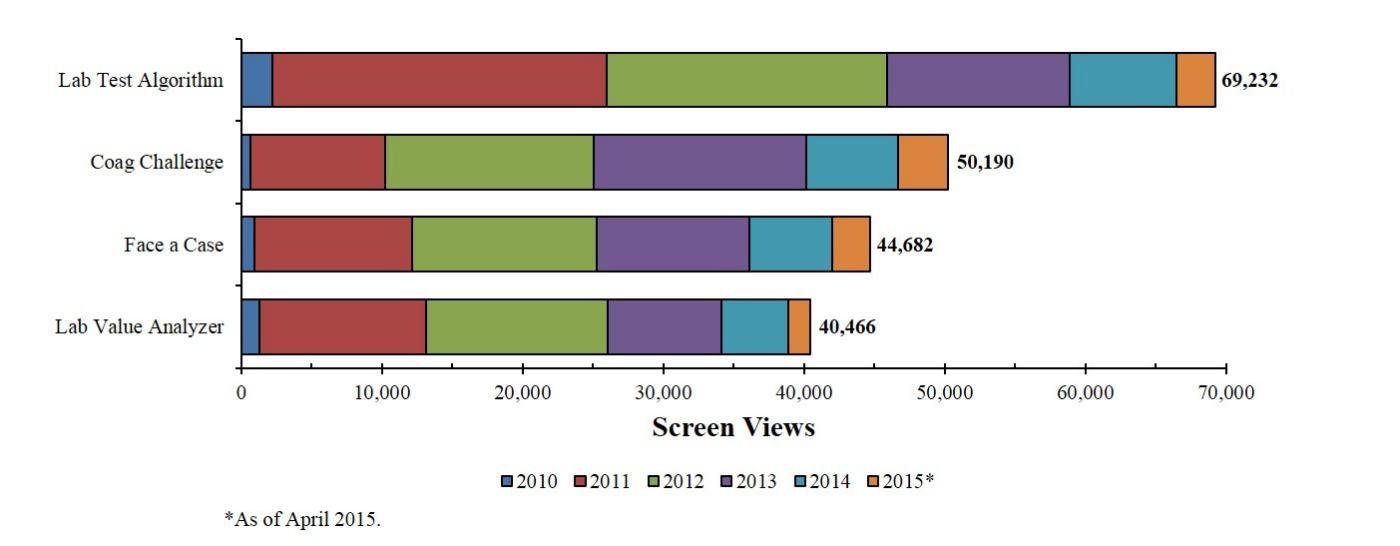
Screen views by app function.

**Figure 3:**
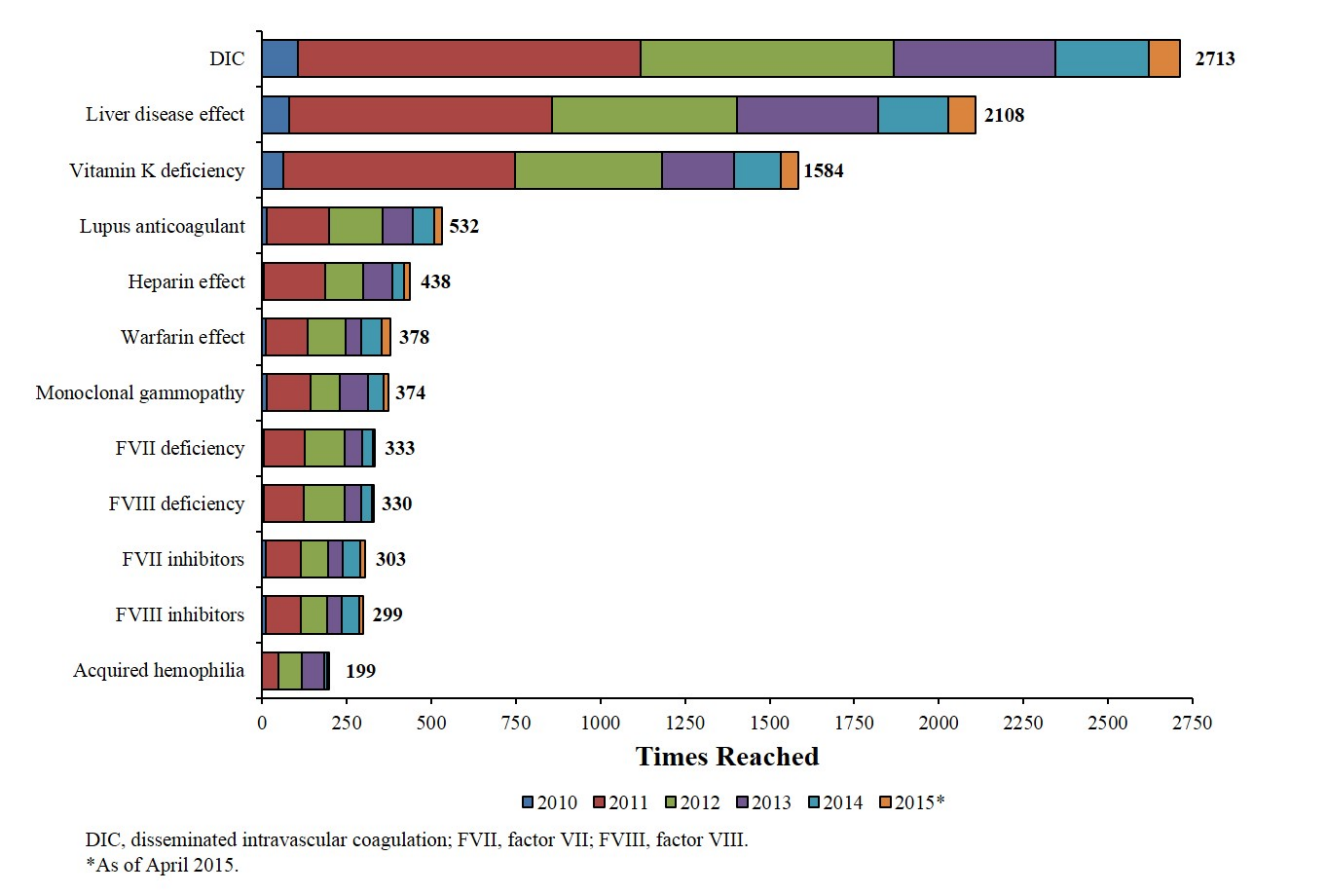
Most common diagnostic endpoints reached.

Numbers of screen views were largely consistent with rates of registered user specialties and degrees or positions, with highest numbers tracked to hematologists-oncologists and MDs, respectively. Screen views tracked by year were also consistent with user specialties; as of April 2015, the specialties accounting for the greatest numbers of screen views were hematology-oncology (99,390 views), hematology (47,808 views), emergency medicine (23,121 views), and internal medicine (22,586 views; Figure 1). More screen views were associated with the Lab Test Algorithm (69,232 views) and Coag Challenge (50,190 views) tools than with the Face a Case (44,682 views) and Lab Value Analyzer (40,466 views) tools (Figure 2). The most common diagnostic endpoints reached were DIC (2713 times), liver disease effect (2108 times), and vitamin K deficiency (1584 times; Figure 3). VWD was reached 62 times.

### Survey Data

Of 3424 Coags Uncomplicated app users who were asked to take the survey, 262 responded (7.65%). Most respondents (71%) were physicians in direct clinical patient care (Table 2); the majority of these were specialized in hematology-oncology (39%) or emergency medicine (21%) and were board-certified (79%). Most physicians (64%) worked in a hospital-based practice setting, and 33% had an office-based practice setting. The average age of respondents was 46 years (range 23-83 years).

**Table 1:** Registered user composition and total screen views.

Specialty and degree or position		Registered users (N=7596) n (%)	Total screen views^a^ n (%)
**Specialty**			
	Hematology-oncology	1534 (20.19)	118,527 (28.20)
	Hematology	1014 (13.35)	90,852 (21.62)
	Emergency medicine	1222 (16.09)	47,312 (11.26)
	>Internal medicine	543 (7.15)	24,769 (5.89)
	Critical care	423 (5.57)	16,332 (3.89)
	Pediatrics	260 (3.42)	10,682 (2.54)
	Surgery	190 (2.50)	7356 (1.75)
	Geriatrics	131 (1.72)	5368 (1.28)
	Hospitalist	108 (1.42)	N/A^b^
	Obstetrics/gynecology	79 (1.04)	2773 (0.66)
	Other	1730 (22.78)	96,336 (22.92)
	Unspecified	362 (4.77)	N/A
**Degree or position**			
	Doctor of medicine	4082 (53.74)	221,484 (51.91)
	Doctor of osteopathy	364 (4.79)	19,431 (4.55)
	Registered nurse	639 (8.41)	28,771 (6.74)
	Nurse practitioner	415 (5.46)	22,067 (5.17)
	Doctor of pharmacy	283 (3.73)	19,332 (4.53)
	Physician assistant	224 (2.95)	16,884 (3.96)
	Social worker	12 (0.16)	N/A
	Other	1569 (20.66)	98,702 (23.13)

^a^As of February 2016.

^b^N/A: not available.

**Table 2:** Survey respondent composition.

Position and physician specialty		Respondents (%) N=262
**Position^a^**		
	Physician in direct patient care practice	71
	Nurse	9
	Academia/professor/instructor	7
	Pharmacist	4
	Physician assistant	4
	Laboratory director	3
	Pathologist	2
	Medical director	2
	Clinical researcher	2
	Patient/parent/caregiver	2
	Laboratory manager/supervisor	2
	Blood bank manager/director	2
**Physician specialty^b^**		
	Hematology-oncology	39
	Emergency medicine	21
	Hematology	7
	Internal medicine	7
	Critical care	6
	Primary care	5
	Hospitalist	3
	Oncology	2
	Pediatrics	2
	Surgery	1
	Obstetrics-gynecology	1

^a^Respondents could select multiple choices.

^b^Of physician respondents (n=185).

**Figure 4:**
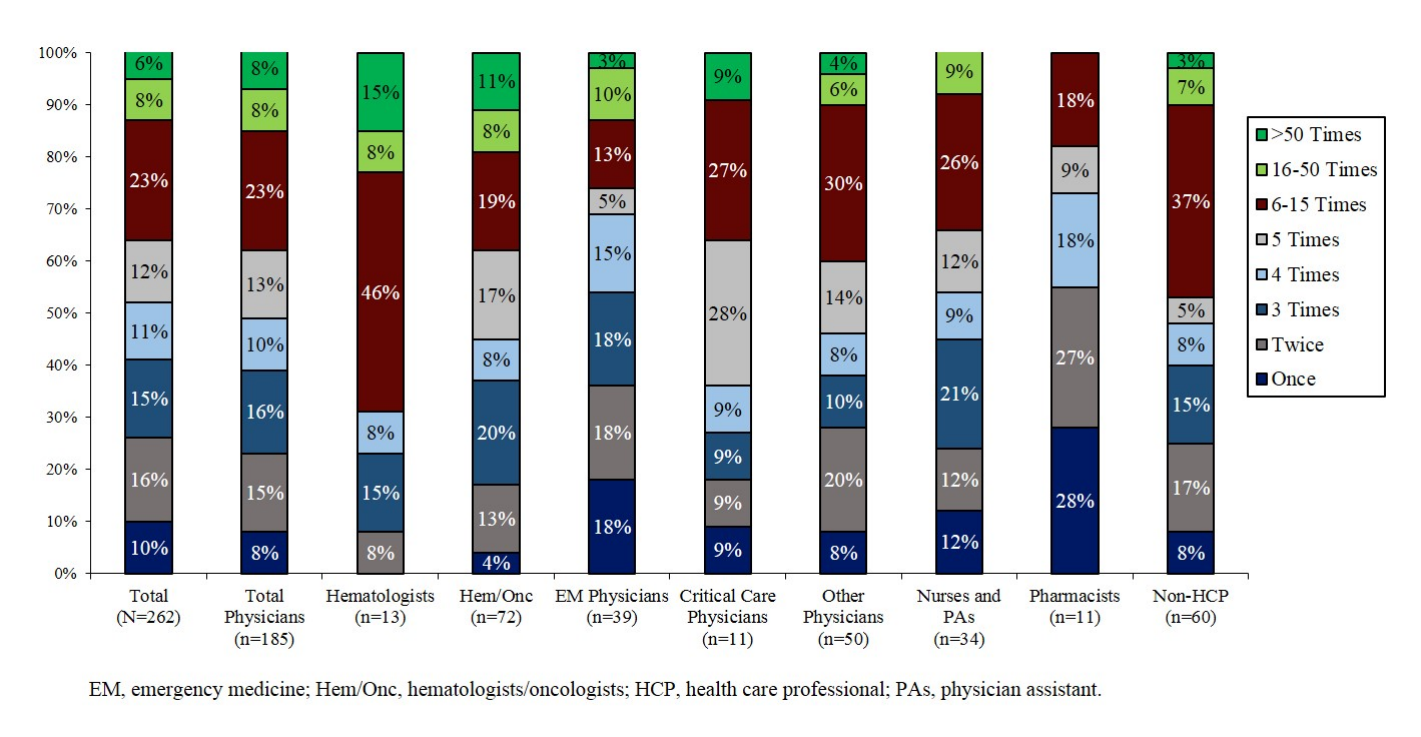
Frequency of app use since downloading.

More than one-third of respondents (37%) reported using the app at least 6 times since downloading it, and some (6%) reported use of more than 50 times (Figure 4). Nearly all respondents had used the Lab Test Algorithm (95%) and Lab Value Analyzer (93%); majorities had also used the Face a Case (73%) and Coag Challenge (65%). The physician specialists reporting the most frequent app use were hematologists (69% used the app at least 6 times) and hematologists-oncologists (38% used the app at least 6 times).

A majority (89.2%) of physicians reported using the app for education related to actual patient cases (eg, point-of-care [POC] education). Among physicians who used the app for education in at least 1 actual patient-case-related instance, the most common circumstances of educational use were related to differential diagnosis (mean 6.35 cases per physician) and to review of educational materials on a disease to confirm a suspected diagnosis (mean 3.46 cases per physician; Table 3). The physician specialties reporting the highest rates of patient-case-related education using the app were hematology (mean 22.36 cases per physician), hematology-oncology (mean 14.06 cases per physician), and critical care (mean 14.00 cases per physician).

Physicians rated both the Lab Value Analyzer (mean 4.43) and Lab Test Algorithm (mean 4.46) tools highly on a 5-point “how helpful” scale (Table 4). On a 5-point scale of likeliness to recommend the app to a colleague or someone with interest in coagulation disorders, most respondents reported a likeliness of 4 (29%) or 5 (57%). Few respondents (7%) reported awareness of a similar app or electronic product.

**Table 3:** Physician use for education to support actual patient cases.

Physician use		Mean number of actual case situations per physician^a^
**By circumstances of educational use**	
	To assist in making the diagnosis	6.35
	To confirm a suspected diagnosis	3.46
	As a teaching aid	1.37
	For case management	0.94
	When specialist not available	0.67
	To decide whether to consult a specialist	0.60
**By app function**	
	Lab Test Algorithm	7.15
	Lab Value Analyzer	6.24
**By physician specialty**	
	Hematology	22.36
	Hematology/oncology	14.06
	Critical care	14.00
	Emergency medicine	12.57
	Other specialties	10.80
**Overall**		13.39

^a^Including only physicians who have used the app for patient-case-related POC education (n=165).

**Table 4:** Helpfulness ratings.

App tool		Mean “how helpful” rating^a^
**Lab Value Analyzer**		
	All physicians	4.43
	Hematologists	4.63
	Hematologists-oncologists	4.36
**Lab Test Algorithm**		
	All physicians	4.46
	Hematologists	4.40
	Hematologists-oncologists	4.40

^a^Rated on a 5-point scale, with 5=very helpful.

## Discussion

### Principal Findings

The goal of developing Coags Uncomplicated was to address educational gaps around the appropriate sequence and interpretation of laboratory tests and to encourage referral to hematology specialists in cases of suspected bleeding disorders. To assess whether the app was reaching the target audience in the United States and whether the types of information sought matched relevant diagnoses and tests, user tracking data were collected and a survey of registered users was performed.

Results of these analyses demonstrated that hematologists and hematologists-oncologists account for the majority of app screen views and report the most frequent app use for POC education to support patient-case-related information about differential diagnostic considerations, disorders, or tests. Most nonhematologists may be less likely to gain awareness of the app and to encounter patients with coagulation disorders, although users specializing in emergency medicine also accounted for high numbers of registered users and screen views. Value of the app among nonhematologists is supported by high “helpfulness” ratings, which were similar between hematologists and physicians overall, and high rates of repeated use across specialties and HCP types.

Due to the high prevalence of use by specialists in hematology and hematology-oncology, who may have variable training and clinical practice experience in benign hematology (bleeding and clotting disorders, compared with malignant disorders), the survey was designed to explore the reasons why users were turning to Coags Uncomplicated, and to identify whether the app is being used more as an educational resource in clinical situations or as a teaching tool. Overall, survey data and app tracking metrics describe app use among physicians and other HCPs in a pattern consistent with value as a clinical POC educational tool. Reported use in patient-case-related circumstances to quickly review relevant educational materials about making a differential diagnosis, to review educational materials on a disease to confirm a suspected diagnosis, and as a teaching aid supports a practical value for filling unmet educational needs and suggests that use of the app may facilitate rapid and efficient differential diagnosis. A majority of physician app users reported using the app to support actual patient-case-related educational needs, suggesting high practical utility and less frequent use as a teaching tool. Additionally, most physician app users worked in a hospital-based practice setting, indicating that most frequent use may occur in the acute care setting.

Data obtained from the survey also assessed the tools that were used most frequently within the app. Whereas a large majority of survey respondents reported having used the Lab Value Analyzer (93%), this tool was associated with the lowest number of screen views. The Lab Value Analyzer was developed largely to help hematology-oncology specialists and those without easy access to specialists experienced in the interpretation of laboratory studies that may have been performed as part of a screening profile, and therefore infrequent use may suggest limited use for these purposes. This pattern of use is also consistent with lower use of the Lab Value Analyzer than the Lab Test Algorithm in actual patient cases, and suggests that the clinical value of the app may be highest in the early stages of diagnosis (before laboratory tests have been run). The Coag Challenge had the lowest rate of respondent use (65%), but the second highest number of screen views, suggesting high rates of repeated use among a subgroup of users who complete the whole challenge, supporting the value of competitive aspects to reinforce learning in adults.

The data regarding most common diagnostic endpoints reached seem to reflect preferential app use in cases of complex and acutely severe conditions that would be seen by those in an emergency room or hospital situation. For example, the most frequently reached endpoints, DIC, liver disease effect, and vitamin K deficiency, are each complex disorders that vary widely in bleeding symptoms [7-9]. Interestingly, VWD, the most common inherited bleeding disorder [10], is notably absent from the list of most common diagnostic endpoints reached. This infrequent use of the app to diagnose VWD may result from physicians’ relative familiarity with diagnosing this disease, the standardized set of laboratory assessments used for VWD diagnosis, and the potential for VWD to present with relatively mild symptoms that may be observed outside of the acute care setting, resulting in infrequent presentations of severe bleeding associated with undiagnosed VWD [10].

Additional tools that may be considered for future versions of Coags Uncomplicated include use of bleeding scores or bleeding assessment tools (BATs) and standard workups for specific bleeding presentations. General diagnostic tools, such as the International Society on Thrombosis and Haemostasis BAT [11] and the Molecular and Clinical Markers for the Diagnosis and Management of Type 1 (MCMDM-1) VWD Bleeding Questionnaire [12], as well as symptom-specific tools such as the Epistaxis Scoring System [13] and the Menorrhagia-Specific Screening tool [14], are useful as screening tools, particularly for mild bleeding disorders. Additionally, standard protocols for assessing hemostatic parameters in patients presenting with specific symptoms, such as heavy menstrual bleeding or epistaxis, may be useful for physicians to ensure appropriate hemostatic evaluation.

### Conclusions

An analysis of Coags Uncomplicated use among US physicians and other HCPs suggests value as a POC educational tool to support differential diagnosis of bleeding disorders. App tracking metrics and survey responses indicate most frequent use among hematologists, hematologists-oncologists, and emergency physicians, and frequent use for education to support actual patient-case-related circumstances. Patterns of use seem to suggest preferential use in cases of complex and acutely severe conditions, which may be encountered by physicians of various specialties. Because bleeding disorders may be challenging to diagnose for those who are not experienced in performing and interpreting advanced hematologic assessments, app use may facilitate efficient and accurate differential diagnoses, reduce delays to appropriate consultation with hematologists, reduce inappropriate use of therapeutic resources, and ultimately reduce mortality of bleeding patients.

## Appendix

Multimedia Appendix 1 Lab Test Algorithm for abnormal prolonged aPTT (activated partial thromboplastin time) only.

Multimedia Appendix 2 Lab Test Algorithm for abnormal prolonged PT (prothrombin time) only.

Multimedia Appendix 3 Lab Test Algorithm for abnormal prolonged PT ( ) and aPTT (activated partial thromboplastin time).

Multimedia Appendix 4 Lab Test Algorithm for normal PT and aPTT (normal platelet count). aPTT: activated partial thromboplastin time. PT: prothrombin time.

Multimedia Appendix 5 Lab Test Algorithm for normal PT and aPTT (decreased platelet count). aPTT: activated partial thromboplastin time. PT: prothrombin time.

Multimedia Appendix 6 Lab Test Algorithm for normal PT and aPTT (increased platelet count). aPTT: activated partial thromboplastin time. PT: prothrombin time.

Multimedia Appendix 7 References supporting the diagnoses and laboratory tests included in Coags Uncomplicated.

## Abbreviations

aPTT: activated partial thromboplastin time
BAT: bleeding assessment tool
DIC: disseminated intravascular coagulation
DO: doctor of osteopathy
HCP: health care professional
MCMDM-1: Molecular and Clinical Markers for the Diagnosis and Management of Type 1 [VWD Bleeding Questionnaire]
MD: doctor of medicine
N/A: not available
NP: nurse practitioner
PA: physician assistant
PharmD: doctor of pharmacy
POC: point-of-care
PT: prothrombin time
RN: registered nurse
VWD: von Willebrand disease
