# Supplementary figures and images for: The Coags Uncomplicated App: Fulfilling Educational Gaps Around Diagnosis and Laboratory Testing of Coagulation Disorders
Source: JMIR Med Educ. 2017 Apr 18;3(1):e6. doi: 10.2196/mededu.6858 (PMC5413799; doi:10.2196/mededu.6858)

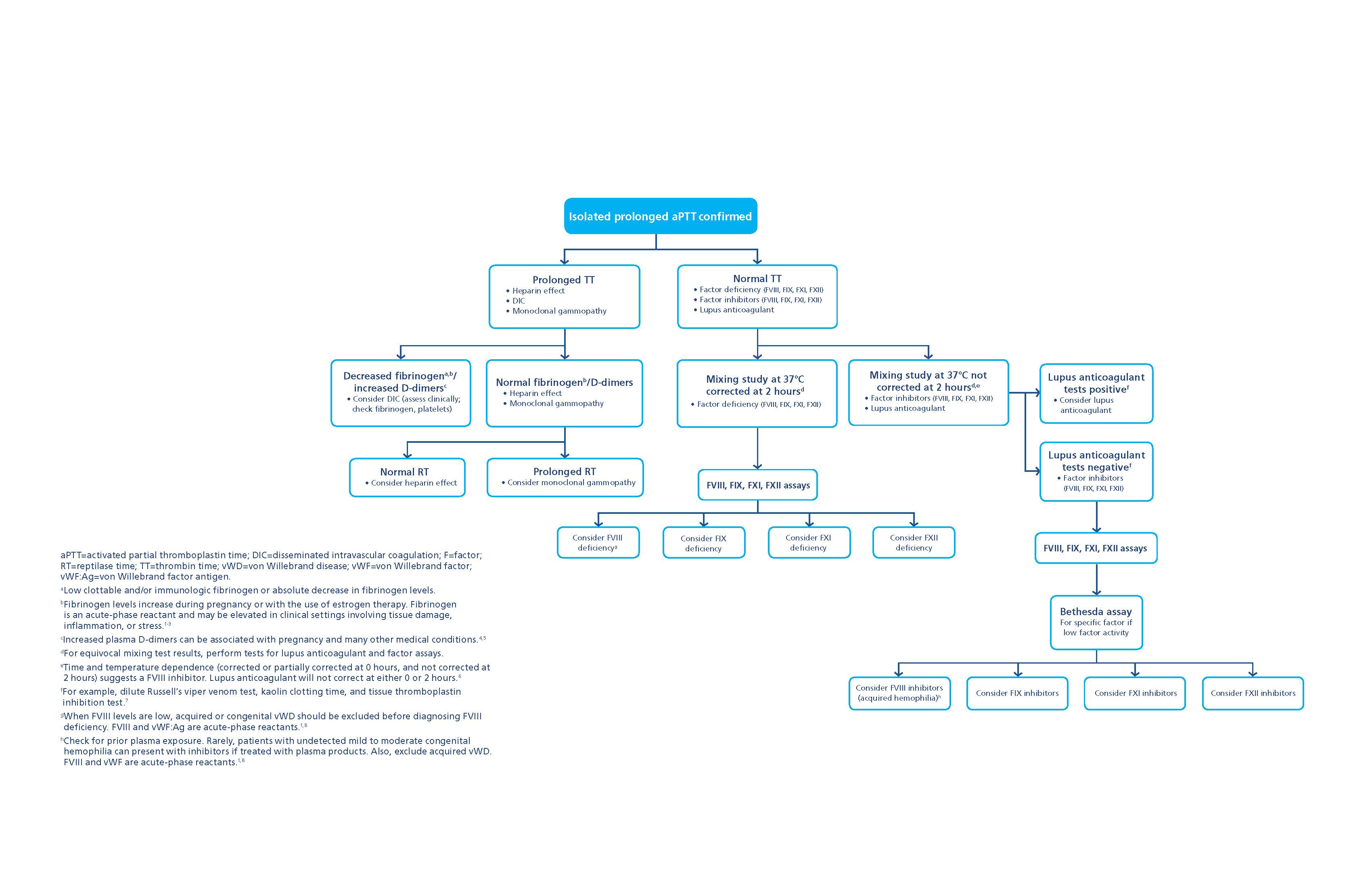

Supplement: Multimedia Appendix 1 [file mededu_v3i1e6_app1.jpg]

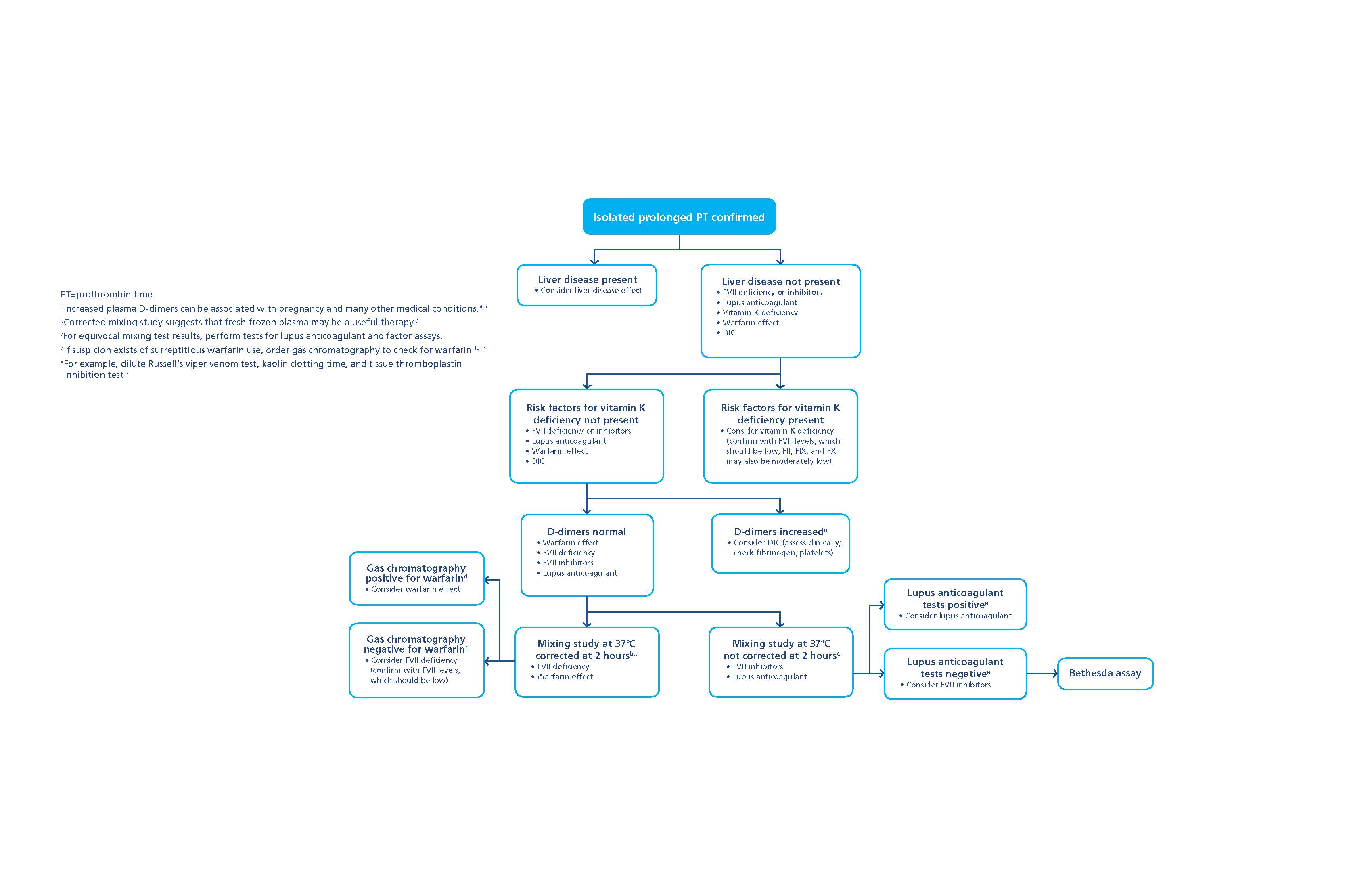

Supplement: Multimedia Appendix 2 [file mededu_v3i1e6_app2.jpg]

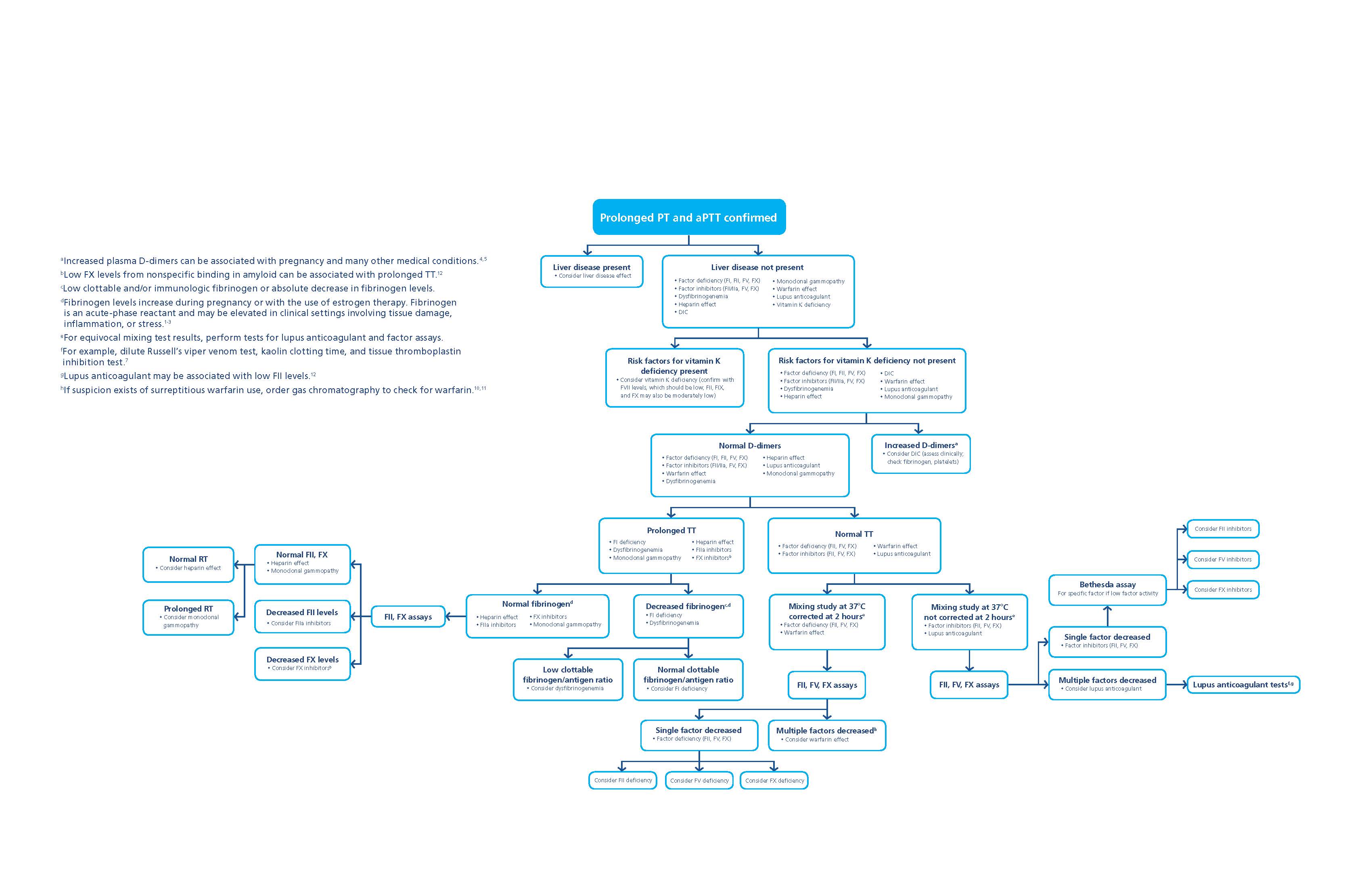

Supplement: Multimedia Appendix 3 [file mededu_v3i1e6_app3.jpg]

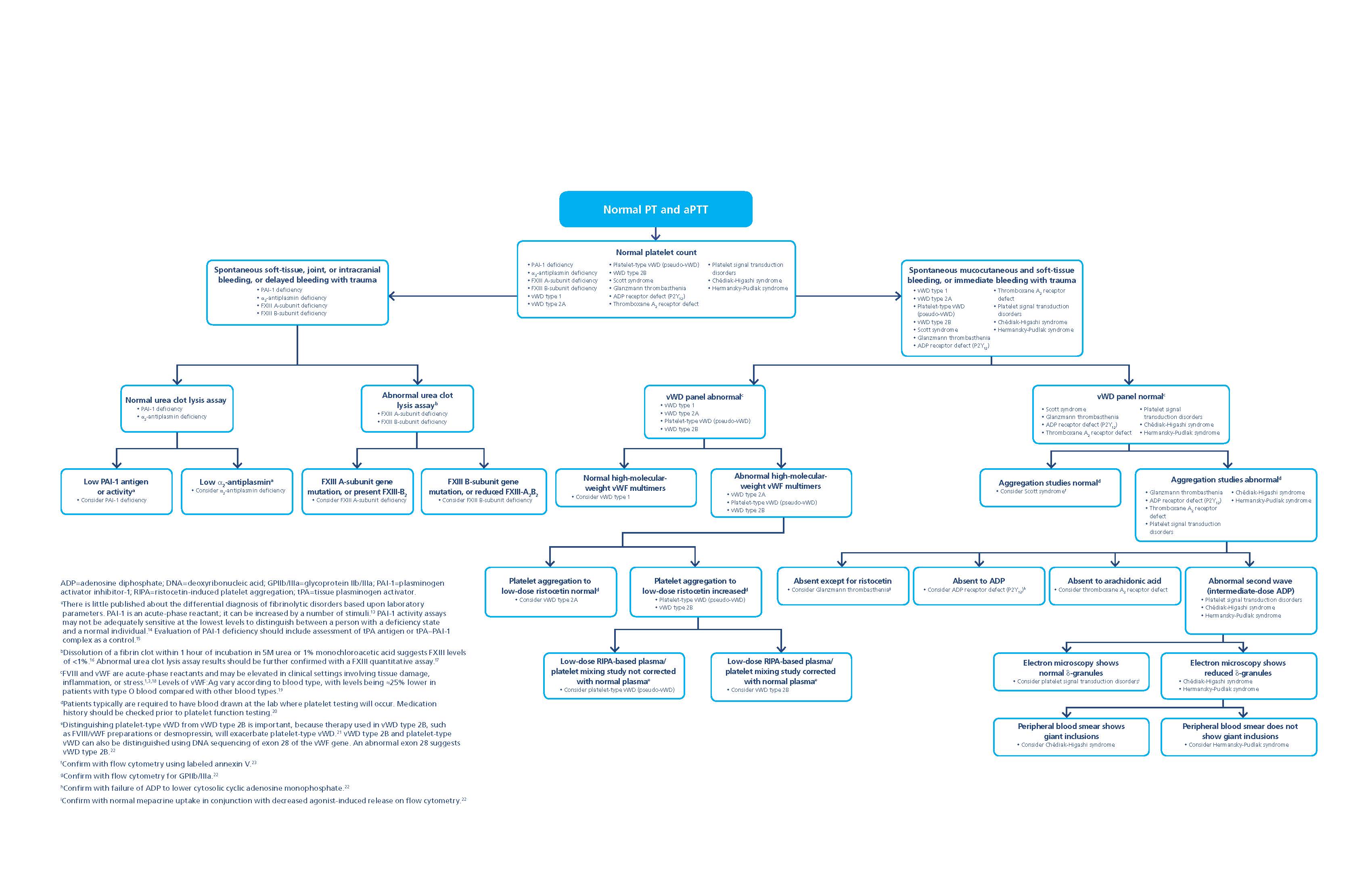

Supplement: Multimedia Appendix 4 [file mededu_v3i1e6_app4.jpg]

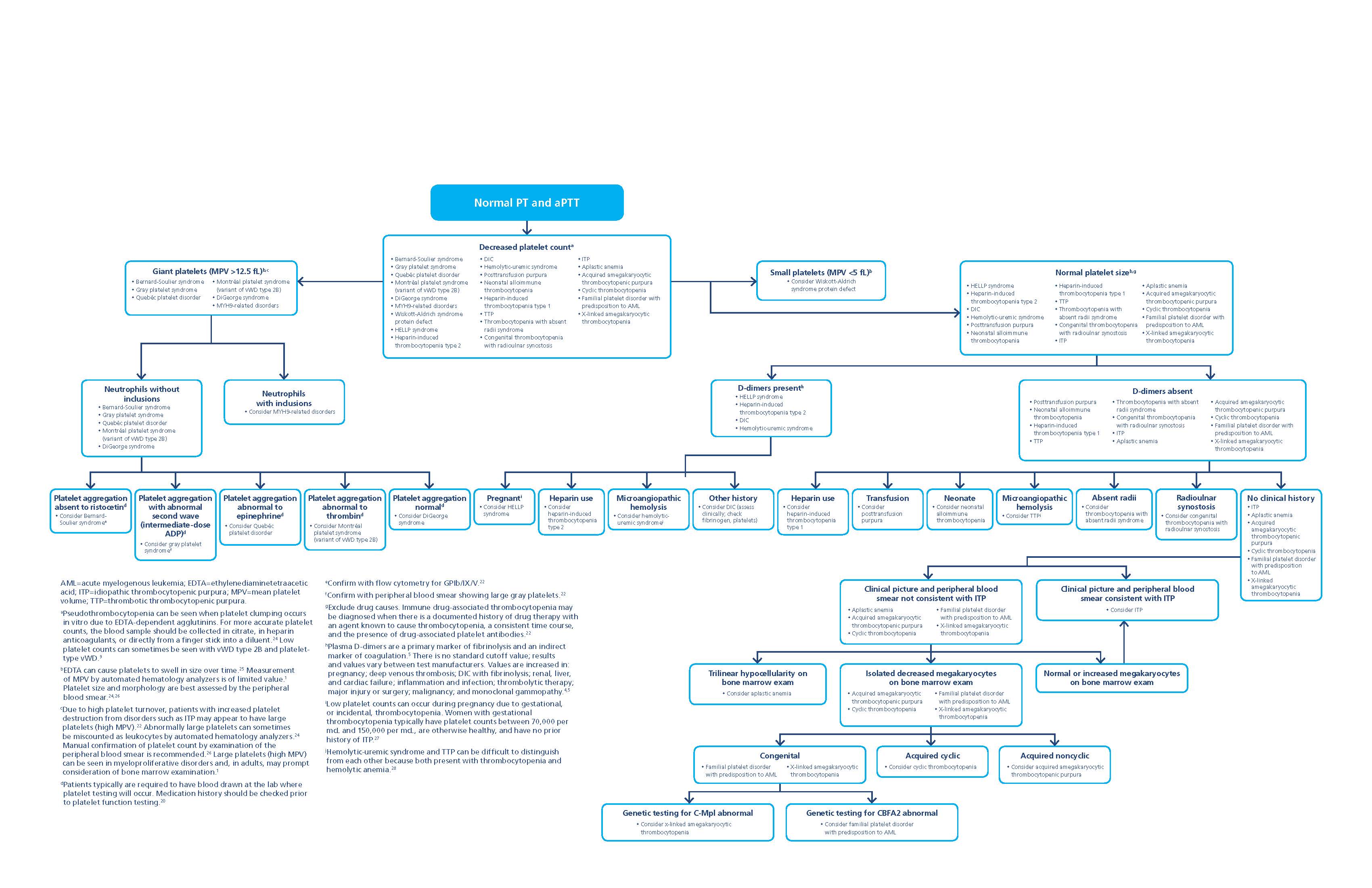

Supplement: Multimedia Appendix 5 [file mededu_v3i1e6_app5.jpg]

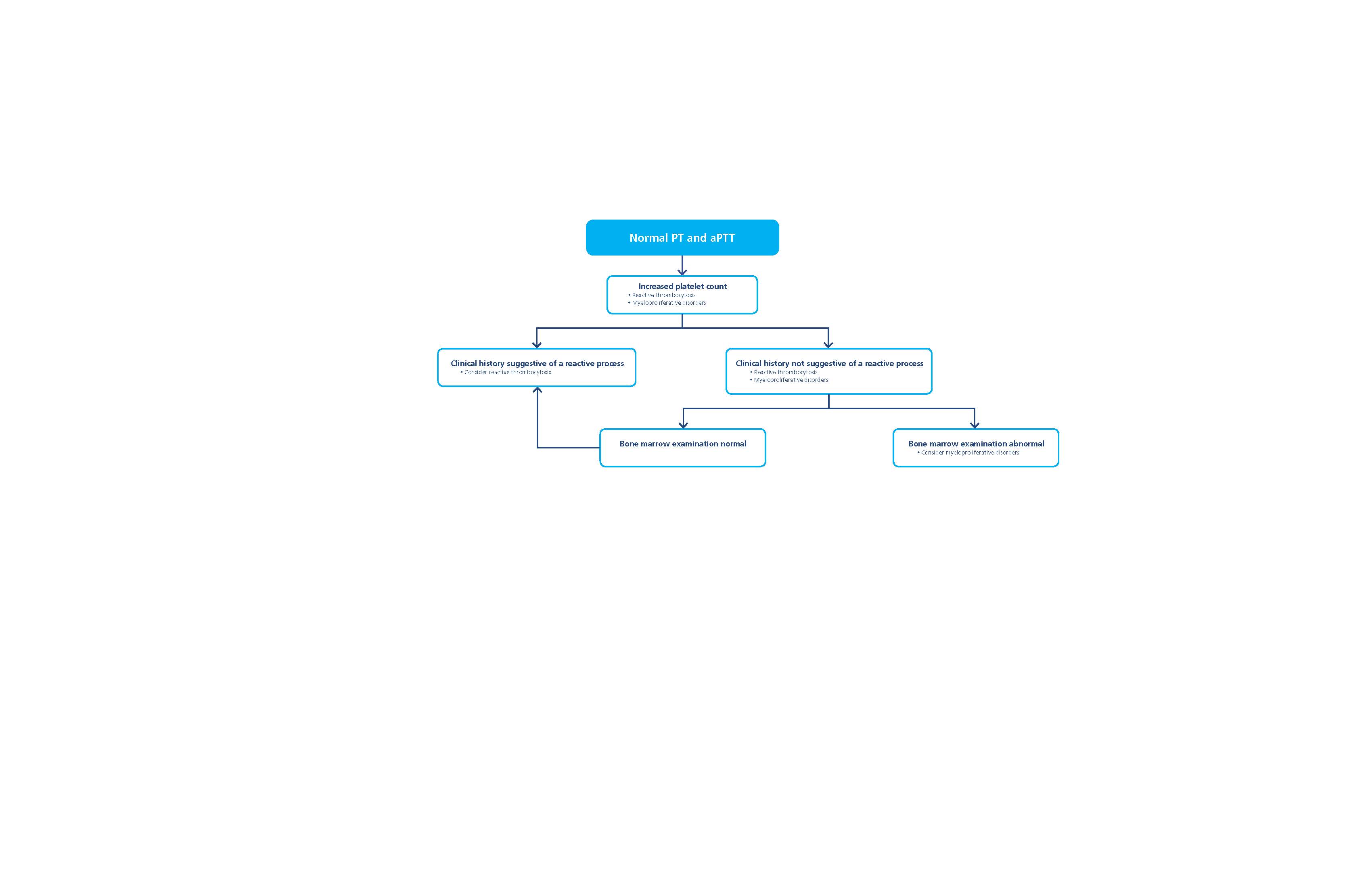

Supplement: Multimedia Appendix 6 [file mededu_v3i1e6_app6.jpg]
