# Supplementary material for: The Coags Uncomplicated App: Fulfilling Educational Gaps Around Diagnosis and Laboratory Testing of Coagulation Disorders
Source: JMIR Med Educ. 2017 Apr 18;3(1):e6. doi: 10.2196/mededu.6858 (PMC5413799; doi:10.2196/mededu.6858)
Supplement: Multimedia Appendix 7 [file mededu_v3i1e6_app7.pdf]

1. Hematology. In: Wallach J, ed. Interpretation of Diagnostic Tests. 8th ed. Philadelphia, PA: Lippincott Williams & Wilkins; 2007:368-560.
2. Bonnar J. Coagulation effects of oral contraception. Am J Obstet Gynecol. 1987;**157**(4, pt 2):1042-1048.
3. Greenberg DL, Davie EW. The blood coagulation factors: their complementary DNAs, genes, and expression. In: Colman RW, Marder VJ, Clowes AW, George JN, Goldhaber SZ, eds. Hemostasis and Thrombosis: Basic Principles and Clinical Practice. 5th ed. Philadelphia, PA: Lippincott Williams & Wilkins; 2006:21-57.
4. Szecsi PB, Jørgensen M, Klajnbard A, Andersen MR, Colov NP, Stender S. Haemostatic reference intervals in pregnancy. Thromb Haemost. 2010;**103**(4):718-727.
5. Respiratory diseases. In: Wallach J, ed. Interpretation of Diagnostic Tests. 8th ed. Philadelphia, PA: Lippincott Williams & Wilkins; 2007:144-170.
6. Konkle BA. Clinical approach to the bleeding patient. In: Colman RW, Marder VJ, Clowes AW, George JN, Goldhaber SZ, eds. Hemostasis and Thrombosis: Basic Principles and Clinical Practice. 5th ed. Philadelphia, PA: Lippincott Williams & Wilkins; 2006:1147-1158.
7. Rand JH, Senzel L. Antiphospholipid antibodies and the antiphospholipid syndrome. In: Colman RW, Marder VJ, Clowes AW, George JN, Goldhaber SZ, eds. Hemostasis and Thrombosis: Basic Principles and Clinical Practice. 5th ed. Philadelphia, PA: Lippincott Williams & Wilkins; 2006:1621-1636.
8. Van Cott EM. von Willebrand disease. In: Kottke-Marchant K, ed. An Algorithmic Approach to Hemostasis Testing. Northfield, IL: College of American Pathologists; 2008:225-235.
9. Schwartz SL, Caruana CC. Disorders of plasma clotting factors. In: Harmening DM, ed. Clinical Hematology and Fundamentals of Hemostasis. 4th ed. Philadelphia, PA: F.A. Davis Company; 2002:495-520.
10. Sunheimer RL, Threatte G, Lifshitz MS, Pincus MR. Analysis: principles of instrumentation. In: McPherson RA, Pincus MR, eds. Henry's Clinical Diagnosis and Management by Laboratory Methods. 21st ed. Philadelphia, PA: Saunders Elsevier; 2007:31-55.
11. Roberts HR, Escobar MA. Less common congenital disorders of hemostasis. In: Kitchens CS, Alving BM, Kessler CM, eds. Consultative Hemostasis and Thrombosis. 2nd ed. Philadelphia, PA: Saunders Elsevier; 2007:61-79.
12. Kessler CM, Acs P, Mariani G. Acquired disorders of coagulation: the immune coagulopathies. In: Colman RW, Marder VJ, Clowes AW, George JN, Goldhaber SZ, eds. Hemostasis and Thrombosis: Basic Principles and Clinical Practice. 5th ed. Philadelphia, PA: Lippincott Williams & Wilkins; 2006:1061-1084.
13. Lazarchick J. Interaction of the fibrinolytic, coagulation, and kinin systems; disseminated intravascular coagulation; and related pathology. In: Harmening DM, ed. Clinical Hematology and Fundamentals of Hemostasis. 4th ed. Philadelphia, PA: F.A. Davis Company; 2002:521-533. 14. Mehta R, Shapiro AD. Plasminogen activator inhibitor type 1 deficiency. Haemophilia. 2008;**14**(6):1255-1260.
15. Chandler WL. Fibrinolytic bleeding disorders. In: Kottke-Marchant K, ed. An Algorithmic Approach to Hemostasis Testing. Northfield, IL: College of American Pathologists; 2008:175-183.
16. Johns CS, Ens GE. Coagulation. In: Harmening DM, ed. Clinical Hematology and Fundamentals of Hemostasis. 4th ed. Philadelphia, PA: F.A. Davis Company; 2002:658-681.
17. Hayes TE. Normal prothrombin time and activated partial thromboplastin time. In: Kottke-Marchant K, ed. An Algorithmic Approach To Hemostasis Testing. Northfield, IL: College of American Pathologists; 2008:169-173.
18. Senzolo M, Burroughs AK. Hemostatic alterations in liver disease and liver transplantation. In: Kitchens CS, Alving BM, Kessler CM, eds. Consultative Hemostasis and Thrombosis. 2nd ed. Philadelphia, PA: Saunders Elsevier; 2007:647-659.
19. Introduction to normal values (reference ranges). In: Wallach J, ed. Interpretation of Diagnostic Tests. 8th ed. Philadelphia, PA: Lippincott Williams & Wilkins; 2007:3-25.

20. Platelet function testing. In: Kitchen S, McCraw A, Echenagucia M, eds. Diagnosis of Hemophilia and Other Bleeding Disorders: A Laboratory Manual. 2nd ed. Montréal, Québec: World Federation of Hemophilia; 2010:124-134. http://wfh.org/2/docs/Publications/Diagnosis_and_Treatment/Lab_Manual2010/Lab_Manual_Nov2010.pdf. Updated September 2011. Accessed October 23, 2012.
21. Othman M. Platelet-type von Willebrand disease: three decades in the life of a rare bleeding disorder. Blood Rev. 2011;**25**(4):147-153.
22. Kottke-Marchant K. Platelet disorders. In: Kottke-Marchant K, ed. An Algorithmic Approach to Hemostasis Testing. Northfield, IL: College of American Pathologists; 2008:185-216.
23. Jobe S, Di Paola J. Congenital and acquired disorders of platelet function and number. In: Kitchens CS, Alving BM, Kessler CM, eds. Consultative Hemostasis and Thrombosis. 2nd ed. Philadelphia, PA: Saunders Elsevier; 2007:139-157.
24. Kottke-Marchant K. Platelet testing. In: Kottke-Marchant K, ed. An Algorithmic Approach to Hemostasis Testing. Northfield, IL: College of American Pathologists; 2008:93-112.
25. Vajpayee N, Graham SS, Bem S. Basic examination of blood and bone marrow. In: McPherson RA, Pincus MR, eds. Henry’s Clinical Diagnosis and Management by Laboratory Methods. 22nd ed. Philadelphia, PA: Saunders Elsevier; 2011:509-535.
26. Blood smear. In: Pagana KD, Pagana TJ, eds. Mosby’s Diagnostic and Laboratory Test Reference. 2nd ed. St. Louis, MO: Mosby-Year Book, Inc; 1995:125-127.
27. Laubach J, Bendell J. Hematologic changes of pregnancy. In: Hoffman R, Benz EJ Jr, Shattil SJ, et al, eds. Hematology: Basic Principles and Practice. 5th ed. Philadelphia, PA: Churchill Livingstone Elsevier; 2009:2385-2396.
28. Moake JL. Thrombotic thrombocytopenic purpura. In: Kitchens CS, Alving BM, Kessler CM, eds. Consultative Hemostasis and Thrombosis. 2nd ed. Philadelphia, PA: Saunders Elsevier; 2007:405-420.
